# Pre- and Postoperative Health Status of Patients with Nonfunctioning and Secretory Pituitary Adenomas and an Analysis of Related Factors

**DOI:** 10.1155/2020/4056591

**Published:** 2020-04-20

**Authors:** Yi Zhang, Xiaopeng Guo, Lijun Wang, Jinzhu Guo, Haiyan Zhao, Shuang Sun, Yanxia Sun, Dongrui Xu, Zihao Wang, Lu Gao, Ming Feng, Bing Xing

**Affiliations:** ^1^Neurosurgery, Peking Union Medical College Hospital, Chinese Academy of Medical Sciences and Peking Union Medical College, Beijing, China; ^2^Key Laboratory of Endocrinology of the Ministry of Health, Peking Union Medical College Hospital, Chinese Academy of Medical Sciences and Peking Union Medical College, Beijing, China; ^3^China Pituitary Disease Registry Center, Beijing, China; ^4^China Pituitary Adenoma Specialist Council, Beijing, China

## Abstract

**Purpose:**

To identify the characteristics of the physical and mental health status of patients with pituitary adenomas, explore the postoperative reversibility of impaired health status, and assess the impact of clinical characteristics, hormone levels, anxiety, depression, and disease stigma on health status.

**Methods:**

We prospectively enrolled 147 and 138 patients with nonfunctioning and secretory pituitary adenomas, respectively. Health status was evaluated in 8 domains using the 36-item Short-Form Health Survey before and 3 months after transsphenoidal surgery. The Self-Rating Anxiety Scale, the Self-Rating Depression Scale, and the Stigma Scale for Chronic Illness were used to assess the psychological status.

**Results:**

Compared with the healthy population reference values, general physical and mental health, social functioning, and role limitations due to physical and psychological health problems were all found to be significantly impaired in the adenoma patients. Health status was worse in patients with adrenocorticotropic hormone- (ACTH-) secreting and growth hormone- (GH-) secreting adenomas than in patients with nonfunctioning adenomas. Among the patients, 11.6% had anxiety and 30.9% had depression. Higher scores for anxiety, depression, and disease stigma; older age; higher body mass index; and tumor recurrence were independent risk factors for health status impairment in at least one domain. Physical function impairment and role limitations caused by physical health problems became worse after surgery, whereas the mental component of health status remained the same.

**Conclusion:**

Health status was impaired in patients with pituitary adenomas, especially secretory adenomas. Physical function and role limitations were worse 3 months after surgery than before surgery. Mental problems, old age, obesity, and tumor recurrence reduced health status.

## 1. Introduction

Pituitary adenomas arise from the anterior pituitary gland and are the second most common primary central nervous system tumors [1]. According to the hormone-secreting function of the tumor cells, pituitary adenomas are divided into two categories: nonfunctioning adenomas and secretory adenomas [2]. Clinical presentations of patients with pituitary adenomas include the following: (1) tumor mass effects—headache, blurred vision, visual field loss, and secondary hypopituitarism, and (2) symptoms of hormone hypersecretion that vary according to the secretory characteristics of different types of tumor cells—acromegalic growth, Cushing's syndrome, infertility, menstrual disorder, palpitation, obstructive sleep apnea/hypopnea, etc. [2–4]. The aforementioned manifestations contribute to body-related inconvenience, mental burdens, limitations in physical and social roles, and impairments in health status and quality of life.

Several previous studies focused on the quality of life of patients with pituitary adenomas [5–9]. Patients with active disease and severe headache, as well as younger patients, had an elevated risk of diminished quality of life [8, 9]. High levels of prolactin (PRL) and 24 h urine free cortisol (24 h UFC) were associated with reduced quality of life in patients with prolactinomas and adrenocorticotropic hormone- (ACTH-) secreting adenomas [8]. After treatment, with the elimination of tumor mass effects and the mitigation of elevated hormone levels, the reduced quality of life in patients with pituitary adenomas was reported to be reversed in some literature [9–11]. However, systemic evaluation was not well achieved. Health-related quality of life, or health status, can be evaluated by the 36-item Short-Form Health Survey (SF-36), which comprises both a physical component and a mental component [12]. In contrast to the disease-specific ACRO-QOL (Acromegaly Quality of Life) [13] and Cushing QoL scales [14], the SF-36 questionnaire is a general scale and can be used for patients with different diseases or even for healthy populations. Therefore, the SF-36 was used in this study to evaluate the health-related quality of life in patients with different adenomas. Tanemura et al. [11] evaluated the health status of 30 patients with nonfunctioning macroadenomas using the SF-36 questionnaire and found that scores on the mental component were increased at 1 month after surgery and remained stable at 6 months, whereas the physical summary scores were initially decreased at 1 month and recovered to normal levels by 6 months after surgery. Fujio et al. [10] demonstrated that mental condition improved at 1 year after surgery in patients with growth hormone- (GH-) secreting adenomas. These studies provided an impression of the health status of patients with pituitary adenomas. In the SF-36, the physical and mental components each assess 4 independent domains. However, changes in the 8 individual domains in patients with pituitary adenomas have not been systematically investigated. Additionally, approximately 30% of patients with pituitary adenomas were reported to have anxiety and depression [15], and we have also noticed that some patients in our clinic feel stigmatized because of their disease, that is, the altered appearance of their face. We were interested in whether these psychological factors had negative effects on patients' health status. In the literature, 6 weeks, 6 months, and 1 year after treatment are used as time points used to evaluate the reversibility of reduced health status [9–11], but the effects at 3 months after treatment, which is also an important time point for surgeons, have not been well evaluated.

Therefore, we prospectively and consecutively enrolled 285 patients with nonfunctioning pituitary adenomas and different types of secretory pituitary adenomas, evaluated all 8 domains of health status using the SF-36 questionnaire, compared the subjects' scores with the healthy population reference values, and then re-evaluated the patients' health status 3 months after surgery. We aimed to characterize the health status of patients with pituitary adenomas, identify the differences among different tumor subtypes, and explore the postoperative reversibility of impaired health status. Additionally, we assessed the levels of anxiety, depression, and disease stigma in the patients and investigated the influence of those factors on patients' health status.

## 2. Materials and Methods

### 2.1. Study Subjects

We prospectively and consecutively enrolled patients with nonfunctioning and secretory pituitary adenomas who were admitted to the Neurosurgery Department at Peking Union Medical College Hospital from January 2018 to June 2018. Secretory pituitary adenomas in this study included GH-secreting adenoma, ACTH-secreting adenoma, prolactinoma, thyroid-stimulating hormone (TSH) secreting adenoma, and follicle-stimulating hormone (FSH) secreting adenoma. The control values were defined as the SF-36 scores of the healthy population (i.e., the reference values).

The inclusion criteria for patients with nonfunctioning adenomas were as follows: (1) a pituitary adenoma was evident on magnetic resonance imaging (MRI) [2] and (2) pituitary-related hormones were within the reference range. Patients who had been treated with hormone replacement therapy before enrollment and had normal hormone levels were also included. The criteria for patients with secretory adenomas were as follows: (1) a pituitary adenoma was evident on MRI and (2) at least one hormone was above the normal range and in accordance with the endocrine diagnostic standard for the corresponding secretory pituitary adenoma [2, 16–19]. The exclusion criteria were as follows: (1) the patient refused to participate in the study, (2) the patient had difficulty finishing the evaluation, and (3) there were potential tumors in other organs that could have influenced the evaluation of health status.

This study was approved by the Institutional Review Board at Peking Union Medical College Hospital, Chinese Academy of Medical Sciences. Each patient signed a written informed consent document before enrollment.

### 2.2. Study Design

Contrast-enhanced MRI of the sellar region was performed, and pituitary-related hormones were assessed in all patients. The indexes and methods for MRI and hormone analysis have been described in our previously published studies [20, 21]. The SF-36 questionnaire [12] was used to assess the health status of all patients the day before surgery. We also recorded patients' general clinical data, including sex, age at diagnosis, body mass index (BMI), marital status, yearly income, number of hospitalizations before admission, previous medical history (including hypertension, diabetes, and heart disease), and educational status. For pituitary-related hormones, we documented GH and insulin-like growth factor 1 (IGF-1) in patients with GH-secreting adenomas and serum cortisol, ACTH, and 24 h UFC in patients with ACTH-secreting adenomas. Evaluation of psychological status, including anxiety, depression, and disease stigma, was also performed before surgery.

After all these procedures, tumor resection was performed through the transsphenoidal approach for each patient. All tumor specimens were pathologically confirmed as pituitary adenomas. The primary treatment for the patients with prolactinomas is the medical treatment with a dopamine agonist. Therefore, in this study, all the enrolled patients with prolactinomas were confirmed to have unsatisfactory responses to bromocriptine with a maximal tolerable dose for more than 3 months before recruitment, in accordance with the consensus [18]. Three months after tumor resection, all patients were required to return to the clinic for disease reassessment, and the SF-36 questionnaire was administered again.

### 2.3. Evaluation of Health Status

We used the SF-36 questionnaire for health status evaluation, which indicated patients' perceptions of physical, emotional, and social health. The SF-36 addresses 8 domains of health: physical functioning (PF), role limitations due to physical health problems (RP), bodily pain (BP), general health perceptions (GHP), social functioning (SF), general mental health (MH), role limitations due to emotional problems (RE), and vitality in terms of energy or fatigue (VT). These domains are usually summarized into two components: PF, RP, BP, and GHP are classified as physical health status, while SF, MH, RE, and VT are classified as mental health status. Possible scores in each domain range from 0 to 100, where higher scores indicate better health status.

### 2.4. Evaluation of Psychological Status

Three questionnaires, namely, the Self-Rating Anxiety Scale [22], the Self-Rating Depression Scale [23], and the Stigma Scale for Chronic Illness [24], were used to evaluate patients' levels of anxiety, depression, and disease stigma, respectively. Each questionnaire provided quantitative scores, and the methods for applying these scales were the same as in the literature [22–24]. If the patients scored over 50 points on the Self-Rating Anxiety Scale, they were considered to have anxiety. Likewise, the patients were considered depressed if they scored over 50 on the Self-Rating Depression Scale. Higher scores on the Stigma Scale for Chronic Illness indicate a higher severity of stigma; there was no cutoff value to distinguish patients with particular levels of stigma.

### 2.5. Statistical Analysis

SPSS version 17.0 (SPSS Inc., IBM, USA) was used to analyze the data. Levene's test was used to evaluate the distribution of the data. Student's *t*-test was used to assess the differences between quantitative data with normally distributed values, and the Mann–Whitney test was used with variables lacking normal distributions. The results are shown as the number or the mean ± standard deviation. The nonparametric variables were reported as median and interquartile range (25th percentile and 75th percentile). Linear regression was used to analyze the correlation between sets of quantitative values. Statistical significance was defined as *p* < 0.05.

## 3. Results

### 3.1. Study Subjects and Clinical Information

A total of 285 patients with pituitary adenomas were enrolled in this study. Among them, 147 patients had nonfunctioning adenomas and 138 patients had secretory adenomas. In the secretory adenoma group, GH-secreting adenomas (66, 47.8%), ACTH-secreting adenomas (35, 25.4%), prolactinomas (24, 17.4%), TSH-secreting adenomas (8, 5.8%), FSH-secreting adenomas (1, 0.7%), and GH/PRL-secreting adenomas (4, 2.9%) were clinically identified and then confirmed by pathologists using immunohistochemistry. The psychiatric status evaluation showed that 33 patients (11.6%) had anxiety and 88 patients (30.9%) had depression. The average score of disease stigma was 35.6 ± 14.0 points. Detailed population data are summarized in Table 1.

### 3.2. Health Status of Patients Compared with the Healthy Population

In patients with pituitary adenomas, the RP, GHP, SF, MH, and RE scores were significantly lower than the healthy population reference values, whereas the VT and BP scores were higher. No difference in the PF scores was detected between the two groups (Table 2). We then compared the SF-36 scores between the reference sample and patients with different subtypes of pituitary adenomas. For the RP, GHP, SF, MH, and RE domains, the scores of patients with nonfunctioning adenomas, GH-secreting adenomas, and ACTH-secreting adenomas were all significantly lower than the reference values. For both the VT and BP domains, the scores of patients with nonfunctioning adenomas were significantly higher than those of the healthy population (*p* < 0.001), whereas no changes were found in the other subtypes. For the PF domain, the scores of patients with nonfunctioning adenomas (*p*=0.043) and prolactinomas (*p*=0.003) were higher than the reference values, but the opposite was true of patients with ACTH-secreting adenomas (*p*=0.012); the combination of these results was consistent with an average PF score similar to the reference value in the overall patient sample.

### 3.3. Health Status of Patients with Nonfunctioning and Secretory Pituitary Adenomas

Compared with the nonfunctioning adenoma group, the group of patients with ACTH-secreting adenomas had lower scores in all 8 domains of the SF-36 questionnaire (all *p* < 0.05), and the group of patients with GH-secreting adenomas had lower scores in 7 domains, that is, all except the PF domain. Interestingly, the scores in the PF (*p*=0.011) and SF (*p*=0.021) domains were significantly lower in patients with ACTH-secreting adenomas than in the patients with GH-secreting adenomas (Table 3).

### 3.4. Factors Related to Health Status

All recorded clinical factors were analyzed to detect correlations with the health status scores. The results showed that the quantitative scores of anxiety, depression, and disease stigma were all significantly correlated with all 8 domains of the SF-36 questionnaire in patients with pituitary adenomas (all *p* < 0.001). Other clinical factors were correlated with one or more domains (Table 4). Multiple regression was performed to uncover independent risk factors for decreased health status. We found that higher scores for anxiety, depression, and disease stigma; higher age; higher BMI; tumor recurrence; and previous medical history were all independent risk factors for health status impairment in at least one domain in patients with pituitary adenomas (Table 5).

### 3.5. Postoperative Improvement in Health Status in Patients with Pituitary Adenomas

Of the 248 patients with nonfunctioning, GH-secreting, and ACTH-secreting adenomas, 212 patients completed the SF-36 questionnaire 3 months after surgery. The results showed that the physical component of health status changed after surgery, whereas the mental component remained the same. Scores in the PF and RP domains were significantly decreased 3 months after surgery, whereas the GHP scores significantly increased (Table 6). In the nonfunctioning adenoma group, postoperative PF (*p*=0.005) and RP (*p*=0.049) scores were lower than those before surgery. In the GH-secreting adenoma group, the PF scores were lower (*p*=0.040) and GHP scores were higher (*p*=0.020) than the preoperative scores. The scores in all 8 domains of the SF-36 in the patients with ACTH-secreting adenomas remained the same 3 months after surgery.

## 4. Discussion

In this study, both the physical and mental health status of the patients with pituitary adenomas, including nonfunctioning adenomas and secretory adenomas, were impaired compared with the healthy population. The patients with secretory adenomas had worse health status than the patients with nonfunctioning adenomas. In the secretory adenoma group, the patients with ACTH-secreting adenomas had the worst health status. This study was the first to explore the impact of anxiety, depression, and disease stigma on patients' health status and to analyze health status changes at 3 months after surgery across different pituitary tumor subtypes. The levels of anxiety, depression, and disease stigma were related to reduced health status scores, and these indexes were independent risk factors for at least one domain of impaired health status. Three months after tumor resection, improvements could be seen in the GHP domain, but the scores for PF and RP became even worse. MH remained at its preoperative level.

Our results showed that the health status evaluated by the SF-36 questionnaire was impaired in patients with pituitary adenomas, which was consistent with the published literature [8–11, 25, 26]. Specifically, after dividing health status into 8 domains, we found that the domains of PF, GHP, MH, RP, and RE were all impaired in patients with pituitary adenomas, whether they were nonfunctioning adenomas, GH-secreting adenomas, or ACTH-secreting adenomas. However, in patients with prolactinomas, only the GHP and MH scores were reduced, and the other indexes were the same as in the healthy population. Surprisingly, the BP and VT were significantly elevated in patients with nonfunctioning adenomas, whereas the patients with other secretory adenomas had scores similar to the reference range. This result was unexpected, and a larger sample size is needed to verify this finding. We could also establish an SF-36 reference range based on the Chinese population and use it as the control in the future.

The patients with different types of pituitary adenomas had different health statuses. The study by Vega-Beyhart et al. [8] showed the SF-36 scores were lower in patients with ACTH-secreting adenomas and prolactinomas than in patients with nonfunctioning adenomas. Our results demonstrated that the patients with GH-secreting adenomas also had lower scores than the patients with nonfunctioning adenomas. Patients with GH-secreting adenomas always have complications of arthritis, heart disease, and respiratory disease [3], and patients with ACTH-secreting adenomas always develop osteoporosis, fractures, and infections [4]. These complications inevitably impair patients' physical and mental health. Therefore, patients with different subtypes of secretory adenomas also have different health statuses. PF, MH, and RP were all more seriously impaired in patients with ACTH-secreting adenomas than in patients with GH-secreting adenomas.

Studies on the risk factors for impaired health status have indicated that headache is related to a reduced SF-36 score, especially the mental component [9, 26]. Levels of 24 h UFC were negatively correlated with mental health status scores but not physical health status scores in patients with ACTH-secreting adenomas [8]. In the present study, we analyzed the relationships between scores and hormone levels, including GH, IGF-1, cortisol, ACTH, and 24 h UFC, but we did not find any significant correlations. The results showed that age, BMI, tumor recurrence, previous medical history, education duration, yearly income, and unemployment were all related to at least one domain of health status. Subsequent multiple regression analyses revealed that age and previous medical history were independent risk factors for impaired PF, BMI was an independent risk factor for poor RP, and tumor recurrence and previous medical history were independent risk factors for impaired GHP.

In this study, the anxiety, depression, and disease stigma scores were all significantly correlated with each of the 8 domains of health status in patients with pituitary adenomas. Higher anxiety, depression, and disease stigma scores were correlated with lower health status scores. The multiple regression results showed that all 8 domains of impaired health status were independently correlated with these psychological problems. Therefore, the diagnosis and treatment of psychological problems in patients with pituitary adenomas should be given more attention. Patients with low levels of anxiety, depression, and disease stigma would have better health status than those with high levels.

Tanemura et al. [11] demonstrated that SF-36 scores initially decreased at 6 weeks after surgery and then increased at 6 months in patients with nonfunctioning adenomas. Another study showed that mental health status was elevated at 1 year after surgical remission in patients with GH-secreting adenomas [10]. In our study, at 3 months after surgery, the domains of PF and RP were even worse than the preoperative values in patients with nonfunctioning adenomas. PF was also worse than its preoperative value in patients with GH-secreting adenomas. However, MH did not change after surgery regardless of tumor type. Thus, in light of our results and the literature, mental health status does not change in the first few months after surgery, whereas physical health status initially worsens in the first 3 months and then shows improvement at 6 months after surgery.

This study had limitations. Although we enrolled 285 patients with pituitary adenomas, the number of patients in each subgroup was not large enough to uncover every positive result. This study did not include a healthy control group but instead used the SF-36 scores of the healthy population (i.e., reference values) as control values. Moreover, long-term follow-up data, that is, 3 to 5 years after surgery, for these patients are not available at this time; we are currently following the patients and will report further results in the future.

## 5. Conclusions

In summary, both physical and mental health status as measured by the SF-36 were impaired in patients with different types of pituitary adenomas. The patients with ACTH-secreting adenomas and GH-secreting adenomas had even worse health status than the patients with nonfunctioning adenomas. PF and RP were worse at 3 months after surgery than before surgery and MH scores remained the same. The patients' levels of anxiety, depression, and disease stigma could significantly worsen both physical and mental aspects of health status. More importance needs to be attached to psychological disorders in patients with pituitary adenomas. Diagnosis and treatment of these disorders could help improve health status and quality of life in patients with pituitary adenomas.

## Figures and Tables

**Table 1:** General data of the patients with pituitary adenomas.

	Patients with pituitary adenomas
Male, *n* (%)	118/41.4%
Age (years)	43.7 ± 14.4
Body mass index (kg/m^2^)	25.8 ± 4.3
Score of anxiety	41.4 ± 8.4
Score of depression	46.3 ± 10.5
Score of disease stigma	35.6 ± 14.0
GH of patients with GH-secreting adenomas (ng/ml)	10.2 (6.2, 31.6)
IGF-1 of patients with GH-secreting adenomas (ng/ml)	827.0 (636.0, 1030.0)
Serum cortisol of patients with ACTH-secreting adenomas (*μ*g/dl)	30.8 (18.1, 37.4)
Serum ACTH of patients with ACTH-secreting adenomas (pg/ml)	77.6 (45.6, 114.8)
24 h UFC of patients with ACTH-secreting adenomas (*μ*g)	519.2 (284.3, 743.9)
Recurrent tumor, *n* (%)	62/21.8%
Macroadenomas, *n* (%)	203/71.2%
Largest diameter of the tumor (cm)	1.6 (0.6, 4.3)
Invasiveness tumor (Knosp >2), *n* (%)	134/47.0%
Visual abnormalities, *n* (%)	46/16.1%
Unmarried, *n* (%)	45/15.8%
Unemployment, *n* (%)	139/48.8%
Period of education (years)	12.3 ± 4.1
Previous medical history, *n* (%)	128/44.9%

GH = growth hormone; IGF-1 = insulin-like growth factor; ACTH = adrenocorticotropic hormone; UFC = urine free cortisol.

**Table 2:** SF-36 scores of patients with pituitary adenomas in comparison with the reference values.

	All patients with pituitary adenomas (*n* = 285)		Nonfunctioning adenomas (*n* = 147)		GH-secreting adenomas (*n* = 66)		ACTH-secreting adenomas (*n* = 35)		PRL-secreting adenomas (*n* = 24)		Reference range
	Score	*p* value	Score	*p* value	Score	*p* value	Score	*p* value	Score	*p* value	
*Physical component*											
PF	85.5 ± 19.2	0.271	87.3 ± 18.3	0.043^*∗*^	85.3 ± 15.8	0.572	71.9 ± 27.7	0.012^*∗*^	92.3 ± 11.8	0.003^*∗*^	84.2 ± 23.3
RP	63.8 ± 42.4	<0.001^*∗*^	71.3 ± 40.3	0.040^*∗*^	62.5 (0, 100)	<0.001^*∗*^	25 (0, 75)	<0.001^*∗*^	100 (75, 100)	0.960	80.9 ± 34.0
BP	78.6 ± 22.5	0.012^*∗*^	82.2 ± 21.9	<0.001^*∗*^	72.5 ± 23.2	0.353	74.6 ± 21.1	0.874	80.2 ± 22.5	0.286	75.2 ± 23.7
GHP	58.0 ± 21.2	<0.001^*∗*^	64.1 ± 20.9	<0.001^*∗*^	49.4 ± 19.4	<0.001^*∗*^	48.4 ± 19.7	<0.001^*∗*^	58.7 ± 18.4	0.002^*∗*^	71.9 ± 20.3

*Mental component*											
SF	74.5 ± 22.3	<0.001^*∗*^	79.1 ± 21.9	0.021^*∗*^	72.0 ± 21.5	<0.001^*∗*^	61.4 ± 21.3	<0.001^*∗*^	75.5 ± 20.4	0.074	83.3 ± 22.7
MH	68.1 ± 18.8	<0.001^*∗*^	71.8 ± 19.0	0.043^*∗*^	64.7 ± 16.6	<0.001^*∗*^	60.6 ± 22.2	0.001^*∗*^	67.2 ± 13.9	0.014^*∗*^	74.7 ± 18.1
RE	62.1 ± 41.9	<0.001^*∗*^	71.9 ± 39.0	0.004^*∗*^	66.7 (0, 100)	<0.001^*∗*^	0 (0, 100)	<0.001^*∗*^	66.7 (66.7, 100)	0.151	81.3 ± 33.0
VT	67.5 ± 20.6	<0.001^*∗*^	73.2 ± 20.1	<0.001^*∗*^	61.2 ± 21.8	0.908	59.3 ± 17.8	0.594	63.8 ± 15.8	0.387	60.9 ± 20.9

PF = physical functioning; RP = role limitations due to physical health problems; BP = bodily pain; GHP = general health perceptions; SF = social functioning; MH = general mental health; RE = role limitations due to emotional problems; VT = vitality-energy or fatigue; GH = growth hormone; ACTH = adrenocorticotropic hormone; PRL = prolactin. *p* values represent comparisons between patients with different types of pituitary adenomas and healthy population reference values. Patients with TSH-secreting adenomas (*n* = 8), FSH-secreting adenomas (*n* = 1), and GH/PRL-secreting adenomas (*n* = 4) were not included in this chart. ^*∗*^*p* < 0.05

**Table 3:** Health status differences among patients with different subtypes of pituitary adenomas.

	Nonfunctioning adenomas	GH-secreting adenomas	ACTH-secreting adenomas	*p*1	*p*2	*p*3
*Physical component*						
PF	87.3 ± 18.3	85.3 ± 15.8	71.9 ± 27.7	0.448	0.003^*∗*^	0.011^*∗*^
RP	71.3 ± 40.3	62.5 (0, 100)	25 (0, 75)	0.002^*∗*^	<0.001^*∗*^	0.008^*∗*^
BP	82.2 ± 21.9	72.5 ± 23.2	74.6 ± 21.1	0.004^*∗*^	0.007^*∗*^	0.656
GHP	64.1 ± 20.9	49.4 ± 19.4	48.4 ± 19.7	<0.001^*∗*^	<0.001^*∗*^	0.808

*Mental component*						
SF	79.1 ± 21.9	72.0 ± 21.5	61.4 ± 21.3	0.029^*∗*^	<0.001^*∗*^	0.021^*∗*^
MH	71.8 ± 19.0	64.7 ± 16.6	60.6 ± 22.2	0.010^*∗*^	0.003^*∗*^	0.290
RE	71.9 ± 39.0	66.7 (0, 100)	0 (0, 100)	<0.001^*∗*^	0.001^*∗*^	0.566
VT	73.2 ± 20.1	61.2 ± 21.8	59.3 ± 17.8	<0.001^*∗*^	<0.001^*∗*^	0.654

PF = physical functioning; RP = role limitations due to physical health problems; BP = bodily pain; GHP = general health perceptions; SF = social functioning; MH = general mental health; RE = role limitations due to emotional problems; VT = vitality-energy or fatigue; GH = growth hormone; ACTH = adrenocorticotropic hormone. *p*1: nonfunctioning adenomas vs. GH-secreting adenomas; *p*2 = nonfunctioning adenomas vs. ACTH-secreting adenomas; *p*3 = GH-secreting adenomas vs. ACTH-secreting adenomas. ^*∗*^*p* < 0.05.

**Table 4:** Relevance of clinical factors for health status in patients with pituitary adenomas.

		PF	RP	BP	GHP	VT	SF	RE	MH
Score of anxiety	*r* value	−0.39	−0.346	−0.482	−0.531	−0.612	−0.438	−0.419	−0.575
	*p* value	<0.001^*∗*^	<0.001^*∗*^	<0.001^*∗*^	<0.001^*∗*^	<0.001^*∗*^	<0.001^*∗*^	<0.001^*∗*^	<0.001^*∗*^

Score of depression	*r* value	−0.353	−0.384	−0.441	−0.507	−0.667	−0.488	−0.454	−0.641
	*p* value	<0.001^*∗*^	<0.001^*∗*^	<0.001^*∗*^	<0.001^*∗*^	<0.001^*∗*^	<0.001^*∗*^	<0.001^*∗*^	<0.001^*∗*^

Score of disease stigma	*r* value	−0.290	−0.375	−0.415	−0.498	−0.512	−0.528	−0.471	−0.519
	*p* value	<0.001^*∗*^	<0.001^*∗*^	<0.001^*∗*^	<0.001^*∗*^	<0.001^*∗*^	<0.001^*∗*^	<0.001^*∗*^	<0.001^*∗*^

Age (years)	*r* value	−0.274	−0.043	−0.014	0.057	0.169	0.076	0.039	0.189
	*p* value	<0.001	0.473	0.812	0.335	0.004^*∗*^	0.198	0.516	0.001^*∗*^

Period of education (years)	*r* value	0.194	0.199	0.078	0.044	0.096	0.066	0.148	−0.641
	*p* value	0.001^*∗*^	0.001^*∗*^	0.187	0.457	0.105	0.265	0.012^*∗*^	0.813

Yearly income (dollars)	*r* value	0.167	0.194	0.051	0.118	0.102	0.096	0.187	0.112
	*p* value	0.005^*∗*^	0.001^*∗*^	0.392	0.047^*∗*^	0.085	0.105	0.002^*∗*^	0.058

Body mass index (kg/m^2^)	*r* value	−0.200	−0.165	−0.088	−0.116	0.017	−0.053	−0.032	0.107
	*p* value	0.001^*∗*^	0.005^*∗*^	0.140	0.051	0.779	0.370	0.593	0.071

Serum cortisol (*μ*g/dl)	*r* value	−0.167	−0.146	−0.170	−0.060	−0.054	−0.142	−0.150	−0.059
	*p* value	0.354	0.416	0.344	0.741	0.764	0.430	0.406	0.742

Serum ACTH (pg/ml)	*r* value	0.075	0.188	0.017	0.050	0.172	0.307	0.155	0.037
	*p* value	0.670	0.280	0.924	0.777	0.324	0.073	0.374	0.834

24 h UFC (*μ*g)	*r* value	0.083	0.156	0.071	0.032	−0.119	0.086	0.212	0.045
	*p* value	0.659	0.395	0.699	0.862	0.517	0.639	0.244	0.806

GH (ng/ml)	*r* value	0.138	−0.084	−0.187	−0.100	−0.190	0.049	−0.195	−0.159
	*p* value	0.365	0.584	0.219	0.512	0.212	0.750	0.200	0.296

IGF-1 (ng/ml)	*r* value	0.066	−0.148	0.024	0.067	−0.081	−0.035	−0.133	−0.145
	*p* value	0.599	0.054	0.847	0.594	0.519	0.783	0.287	0.247

Sex	t/*z* value	1.607	‐0.733	0.361	1.542	0.408	0.172	‐0.583	0.641
	*p* value	0.109	0.464	0.718	0.124	0.683	0.863	0.560	0.522

Unmarried	t/*z* value	−1.648	−0.582	−0.559	−0.997	0.436	0.662	−0.683	1.771
	*p* value	0.100	0.560	0.576	0.320	0.663	0.509	0.494	0.078

Unemployment	t/*z* value	3.287	−1.638	0.929	−0.818	0.927	0.837	−0.593	0.136
	*p* value	0.001^*∗*^	0.101	0.354	0.414	0.355	0.403	0.553	0.892

Recurrent tumor	t/*z* value	−1.862	−0.827	−1.616	−2.245	0.019	−1.994	−1.331	−0.717
	*p* value	0.064	0.408	0.107	0.026^*∗*^	0.985	0.047^*∗*^	0.183	0.474

Previous medical history	t/*z* value	−5.090	−2.629	−0.925	−3.308	−0.045	−1.319	−1.414	−0.426
	*p* value	<0.001^*∗*^	0.009^*∗*^	0.355	0.001^*∗*^	0.964	0.187	0.157	0.670

PF = physical functioning; RP = role limitations due to physical health problems; BP = bodily pain; GHP = general health perceptions; SF = social functioning; MH = general mental health; RE = role limitations due to emotional problems; VT = vitality-energy or fatigue; GH = growth hormone; IGF-1 = insulin-like growth factor 1; ACTH = adrenocorticotropic hormone; UFC = urine free cortisol. ^*∗*^*p* < 0.05.

**Table 5:** Independent risk factors for the health status of patients with pituitary adenomas.

	PF	RP	BP	GHP	VT	SF	RE	MH
Anxiety scores	−0.405^*∗*^	NA	−0.582^*∗*^	−0.730^*∗*^	−0.484^*∗*^	NA	NA	−0.498^*∗*^
Depression scores	NA	−1.182^*∗*^	−0.370^*∗*^	−0.363^*∗*^	−0.856^*∗*^	−0.659^*∗*^	−1.131^*∗*^	−0.722^*∗*^
Disease stigma scores	−0.200^*∗*^	NA	NA	−0.241^*∗*^	−0.163^*∗*^	−0.390^*∗*^	−0.463^*∗*^	−0.190^*∗*^
Age (years)	−0.350^*∗*^	NA	NA	NA	NA	NA	NA	NA
Body mass index (kg/m^2^)	NA	−1.805^*∗*^	NA	NA	NA	NA	NA	NA
Recurrent tumor	NA	NA	NA	−4.729^*∗*^	NA	NA	NA	NA
Previous medical history	−6.855^*∗*^	NA	NA	−6.366^*∗*^	NA	NA	NA	NA

PF = physical functioning; RP = role limitations due to physical health problems; BP = bodily pain; GHP = general health perceptions; SF = social functioning; MH = general mental health; RE = role limitations due to emotional problems; VT = vitality-energy or fatigue. Values in the table represent the B values (after multiple regression) of the clinical factors for health status in patients with pituitary adenomas. NA indicates that the results of the multiple analysis were not significant. ^*∗*^*p* < 0.05.

**Table 6:** Postoperative changes in health status in patients with pituitary adenomas.

	All pituitary adenomas (*n* = 212)			Nonfunctioning adenomas (*n* = 125)			GH-secreting adenomas (*n* = 56)			ACTH-secreting adenomas (*n* = 31)		
	Preop. score	Postop. score	*p* value	Preop. score	Postop. score	*p* value	Preop. score	Postop. score	*p* value	Preop. score	Postop. score	*p* value
*Physical component*												
PF	85.8 ± 19.2	80.2 ± 17.7	0.001^*∗*^	88.4 ± 16.8	82.5 ± 16.2	0.005^*∗*^	85.3 ± 16.1	78.2 ± 19.6	0.040^*∗*^	71.5 ± 28.7	71.5 ± 19.1	1.000
RP	62.6 ± 42.5	54.3 ± 42.4	0.036^*∗*^	69.0 ± 40.2	58.8 ± 41.7	0.049^*∗*^	62.5 (0, 100)	50 (0, 100)	0.698	25 (0, 75)	12.5 (0, 75)	0.770
BP	76.5 ± 24.1	77.0 ± 22.0	0.826	79.4 ± 24.5	79.9 ± 18.9	0.873	70.7 ± 23.9	76.4 ± 23.5	0.204	73.3 ± 23.2	64.0 ± 27.2	0.153
GHP	57.4 ± 21.1	62.9 ± 22.2	0.007^*∗*^	62.8 ± 21.2	66.7 ± 21.7	0.153	49.0 ± 19.5	57.8 ± 20.5	0.020^*∗*^	50.3 ± 19.7	58.0 ± 23.7	0.169

*Mental component*												
SF	66.4 ± 21.2	67.5 ± 21.7	0.174	77.9 ± 21.7	78.8 ± 23.5	0.753	69.9 ± 22.2	76.3 ± 19.9	0.107	62.5 ± 21.9	66.9 ± 25.1	0.461
MH	67.6 ± 19.3	67.7 ± 20.0	0.955	71.6 ± 19.1	71.6 ± 18.9	1.000	63.8 ± 16.8	67.7 ± 17.2	0.224	58.6 ± 22.7	57.2 ± 24.4	0.814
RE	61.2 ± 41.8	61.9 ± 41.0	0.852	70.7 ± 38.5	67.5 ± 38.9	0.514	66.7 (0, 100)	67 (0, 100)	0.159	0 (0, 100)	33 (0, 91.75)	0.776
VT	66.4 ± 21.2	67.5 ± 21.7	0.559	72.0 ± 20.3	72.6 ± 20.0	0.790	59.6 ± 22.0	63.5 ± 19.2	0.327	58.4 ± 19.8	58.4 ± 26.2	1.000

PF = physical functioning; RP = role limitations due to physical health problems; BP = bodily pain; GHP = general health perceptions; SF = social functioning; MH = general mental health; RE = role limitations due to emotional problems; VT = vitality-energy or fatigue; GH = growth hormone; ACTH = adrenocorticotropic hormone; Preop. = preoperative; Postop. = postoperative. *p* values represent comparisons between postoperative and preoperative scores on the SF-36 questionnaire. ^*∗*^*p* < 0.05.

## Data Availability

The data used to support the findings of this study are available from the corresponding author upon request.
